# Applications of Surface-Enhanced Raman Scattering in Biochemical and Medical Analysis

**DOI:** 10.3389/fchem.2021.664134

**Published:** 2021-05-07

**Authors:** Aleksandra Szaniawska, Andrzej Kudelski

**Affiliations:** Faculty of Chemistry, University of Warsaw, Warsaw, Poland

**Keywords:** surface enhanced Raman spectroscopy, DNA, proteins, cells, tissues, cancer

## Abstract

In this mini-review, we briefly describe certain recently developed applications of the surface-enhanced Raman spectroscopy (SERS) for determining various biochemically (especially medically) important species from ones as simple as hydrogen cations to those as complex as specific DNA fragments. We present a SERS analysis of species whose characterization is important to our understanding of various mechanisms in the human body and to show its potential as an alternative for methods routinely used in diagnostics and clinics. Furthermore, we explain how such SERS-based sensors operate and point out future prospects in this field.

## Introduction

Surface-enhanced Raman scattering (SERS) is one of the most sensitive analytical tools known—in some cases, it is possible to record a high-quality SERS spectrum dominated by the contribution of even a single molecule (Kneipp et al., 2008). The SERS spectroscopy is therefore considered a very promising option for routine analytical techniques used in medical, biochemical, environmental, and food analyses.

In this mini-review, we briefly describe certain recently developed applications of SERS spectroscopy for characterizing biochemically (and especially medically) important compounds. We also outline the basic theoretical background of the SERS effect and discuss potential future applications of SERS in this field. We hope that this paper will be useful for researchers who are planning to enter this fascinating field.

## Surface-Enhanced Raman Spectroscopy

In the 1970's, it was observed that the Raman signal generated by molecules adsorbed on some nanostructured materials was increased by many orders of magnitude. This phenomenon (called SERS) was explained as a result of the synergistic cooperation of two mechanisms based on: (i) the excitation of the localized surface plasmons and (ii) the chemical interactions (see Supplementary Material). When nanostructures formed from materials with a negative real and a small positive imaginary dielectric constant at a given excitation frequency (e.g., Au, Ag, and Cu) interact with an electromagnetic wave, the collective oscillations of surface conduction electrons (called surface plasmons) are induced, that generate an additional electric field in close proximity to the illuminated nanostructure. In the case of homogeneous plasmonic nanostructures, the strongest enhancement of the electromagnetic field occurs at the sharp apexes and edges; in a case of agglomerates or aggregates of plasmonic nanostructures, very large field enhancement is observed in the slits between nanograins—such places are called “hot spots.” In the SERS spectroscopy, the increase in the efficiency of the Raman signal generated is roughly proportional to the fourth power of the field enhancement (Aroca, 2006; Kudelski, 2009), and because of this fourth power dependence, very large SERS enhancement factors can be achieved, making the SERS spectroscopy one of the most sensitive analytical tools. The chemical mechanism of SERS involves the hybridization of orbitals of the adsorbed molecules with the orbitals of metal, which facilitates resonance Raman scattering. The chemical mechanism is only important for molecules interacting directly with the metal surface, and therefore, is usually not operating in SERS sensors.

## Surface-Enhanced Raman Scattering for (Bio)Medical Applications

The SERS spectroscopy has been widely used for analyzing various biosamples, including DNA (Pyrak et al., 2019; Zhang et al., 2019a), RNA (Lee et al., 2019; Han et al., 2021), cancer markers (Choi et al., 2020), bacteria (Andrei et al., 2021), viruses (Chen et al., 2020), genes (Vo-Dinh et al., 2002), drugs (Jaworska et al., 2016), pathological markers on cellular membranes and tissues (Wallace and Masson, 2020), other biomolecules, ion concentrations, and redox potential in cells (Jaworska et al., 2021), and even *in vivo* SERS measurements on mice (Wen et al., 2021). In many cases, the results obtained are at a level of detection not achievable by other analytical methods. However, the Raman spectroscopy still remains to be included among the routinely applied biochemical methods, such as UV–VIS spectroscopy, fluorescence, and PCR. Recent experiments demonstrate the huge potential of this technique, and it is possible that at some point the SERS spectroscopy will be successfully (routinely) performed on medical samples. Later, we discuss the “hottest” examples of the SERS applications, divided into two groups: label-free detection with easy sample preparation and complicated data analysis, and SERS-based nanosensors, which very often produce zero-one results.

### Label-Free Direct Detection *via* SERS

**Figure 1:**
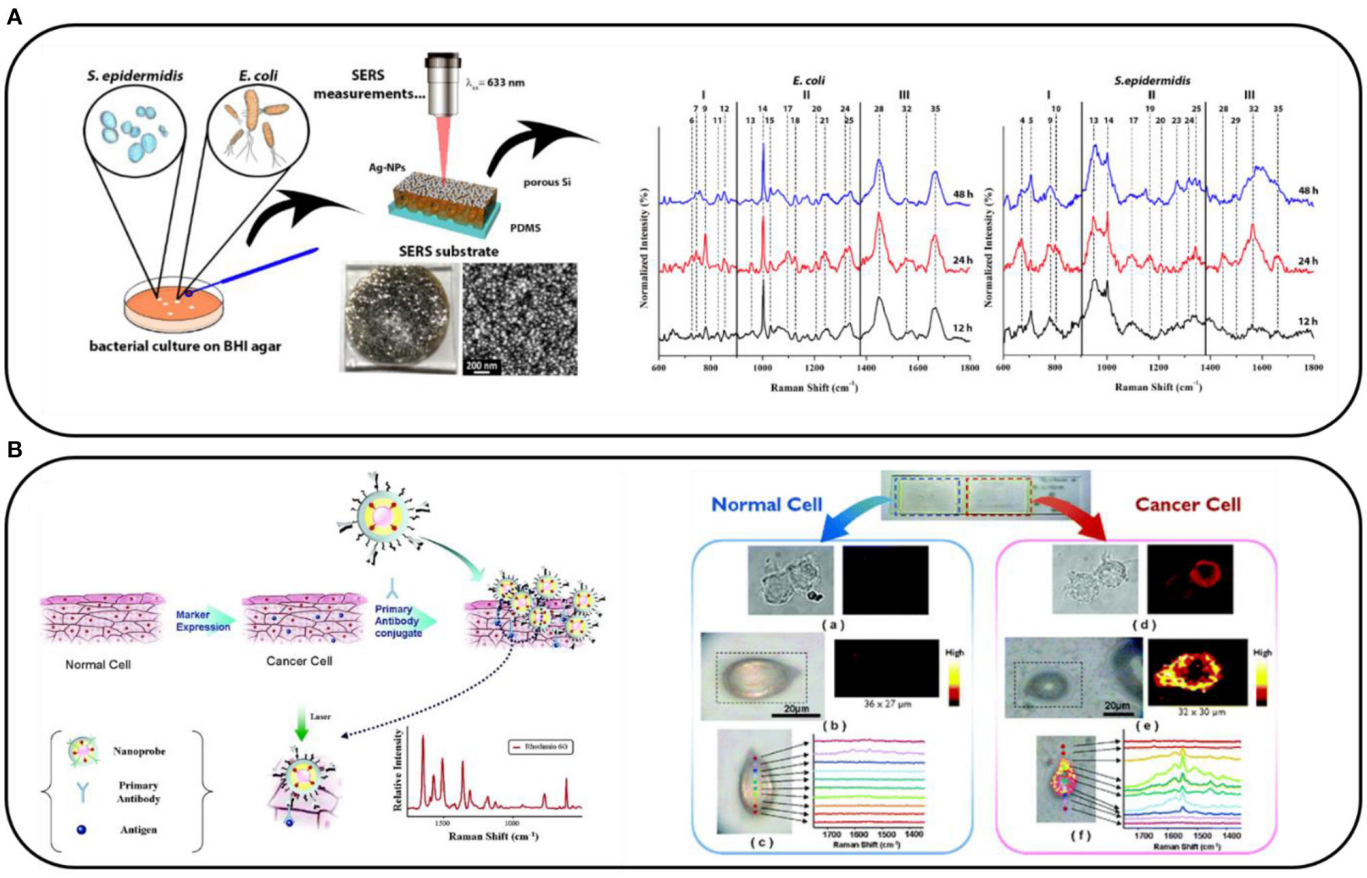
**(A)** Scheme of the label-free surface-enhanced Raman spectroscopy (SERS) analysis of *Escherichia coli* and *Staphylococcus epidermidis* grown on the brain heart infusion (BHI) agar and their SERS spectra collected after 12, 24, and 48 h of culturing. Adapted with permission from Paccotti et al. (2018). **(B)** Schematic diagram depicting the immobilization of Au/Ag core–shell nanoprobes on phospholipase Cγ1 (PLCγ1)-expressing human embryonic kidney (HEK) 293 cells and SERS images of a normal HEK293 cell and a PLCγ1-expressing HEK293 cell. Adapted with permission from Lee et al. (2007). Copyright (2007) American Chemical Society.

The experimental procedure in the label-free SERS detection requires only the adsorption of the analyzed sample directly on the nanostructured plasmonic surface. The obtained spectra are usually, however, difficult to analyze due to the low signal-to-noise ratio and small differences between obtained spectra (e.g., spectra of healthy and cancer cells or different bacteria types). The example of the label-free experiment is shown in Figure 1A. Although this approach is less specific than a nanosensor-based approach, it still provides quite promising results when combined with highly advanced data analysis. Some of the latest examples of label-free SERS applications are: measuring the urine of a patient to detect and monitor the risk of a relapse of prostate cancer (Ma et al., 2021); characterizing the hepatitis C viral (HCV) RNA extracted from different blood samples of patients with HCV infection (Nasir et al., 2020); diagnosing ulcerative colitis in blood plasma (Tefas et al., 2021); and assessing the effectiveness of antiplatelet therapy (Zyubin et al., 2020). In label-free experiments, unfortunately, it is usually impossible to find a new band differentiating two sets of samples. However, by means of chemometrics, we can find subtle differences within the relative intensity or shape of bands. On the one hand, such a classification is not definite, and it is hard to say if this could ever be possible. On the other hand, SERS is one of the most sensitive techniques for detecting compounds, such as neurotransmitters at the attomolar level, and has significant advantages over routine methods (Lee et al., 2021b). The label-free detection of the dengue virus in blood samples is also an impressive example of a fast SERS-based procedure that requires only a very small sample (5 μl) (Gahlaut et al., 2020). The SERS spectra were recorded from samples placed on nanostructured silver substrates; the samples from patients with dengue positive contained dengue-specific immunoglobulin (IgM) antibodies. Also, many bacteria have been detected using a label-free approach. For example, a SERS-active platform based on a polymer nanofiber mat has been shown to be a reliable SERS surface for detecting *Staphylococcus aureus, Pseudomonas aeruginosa*, and *Salmonella typhimurium* in blood plasma at a concentration of 10^3^ colony forming unit/ml (Witkowska et al., 2017). Also, the identification of the cellular composition of Gram-positive and Gram-negative bacteria by using mesoporous silicon-based substrates decorated with silver nanoparticles was carried out, and spectral differences were noticeable regarding their different cell cycles (Paccotti et al., 2018).

In the above-described experiments, SERS proved itself as a reliable methodology. However, the label-free approach is limited by the fact that not all the changes in characteristics of a sample are connected with significant spectral differences. Therefore, in our opinion, the most promising way of introducing SERS into clinics is to prepare sensors that make it possible to detect specific molecules at an ultra-low limit of detection, instead of using the easier but less reliable label-free approach.

### Surface-Enhanced Raman Scattering-Based Nanosensors

An alternative way of using SERS is to prepare nanosensors that contain Raman reporter molecules (RRMs). These are mainly organic dyes such as rhodamine 6G or malachite green, which have an extremely high cross-section for Raman scattering. We can combine these molecules directly with the substance that we want to detect and record the Raman signal of the RRM instead of, for example, the Raman signal of the proteins, which is much lower. An example of such a sensor allowing the distinguishing of over-expressing specific markers of normal and cancer cells can be seen in Figure 1B. Briefly, metal nanoparticles labeled with RRM and specific antibodies interact specifically with the markers expressed on the cellular membrane. The SERS spectra of the cells are recorded (in the form of a map), and the signal from the RRM indicates the presence of the selected marker on the cell (Lee et al., 2007). In this way, Lee et al. used SERS to study human embryonic kidney (HEK) 293 cells expressing phospholipase Cγ1 (PLCγ1). The SERS spectroscopy provided a better quantitative estimation of the signal intensity than the standard fluorescence did. A similar procedure can be applied to many other molecules. Later, we present our subjective choice of the most elegant and promising SERS-based sensors for diagnostic applications.

#### Therapeutic Drug Monitoring

Therapeutic drug monitoring (TDM) involves assessing the drug concentration in a biological matrix (most commonly plasma or serum) at a known time related to administration and interpreting these concentrations in terms of relevant clinical parameters (target range and pharmacokinetics of the drug) (Jaworska et al., 2016). In TDM experiments, the easiest approach is simply to investigate the recorded SERS spectra of the body fluid (urine, blood, and blood plasma) that contains the drug of interest and monitor the intensity of the bands assigned to that drug (Markina et al., 2020). Unfortunately, due to the complexity of the sample, this procedure requires a bit of luck as spectral signatures of the drug are not necessarily seen in the recorded spectra because of the interference from the signals from the body fluid. Therefore, a more common practice is to combine SERS with extraction methods, such as liquid–liquid or solid-phase extraction.

#### Detection of Glucose

Another topic of interest in medical diagnostics is monitoring the level of glucose. Nowadays, this requires several measurements of blood daily, which causes discomfort, pain, and the risk of contamination. Developing a less invasive, continuous glucose monitoring device would have a great impact on 415 million diabetics worldwide. For example, a low-cost SERS sensor for the *in situ* intradermal detection of glucose was developed by Ju et al. (2020). The sensor was calibrated within a range of 0–20 mM in skin phantoms and then successfully tested for the *in vivo* quantification of glucose in a mouse model of streptozotocin (STZ)-induced type I diabetes.

#### Disease Markers

The traditional tissue biopsy is limited in enhancing our understanding of the heterogeneity and dynamic evolution of tumors (Zhang et al., 2019c). Instead, analyzing circulating cancer markers in various body fluids, commonly referred to as “liquid biopsy,” has recently attracted much interest for its great potential in non-invasive early cancer screening, tumor progression monitoring, and therapy response assessment. Thus, blood can also be tested for the presence of circulating tumor cells (CTCs), which play a crucial role in tumor metastasis. CTCs are epithelial cancer cells that have fallen from solid tumors into the bloodstream, and which, after circulating in the blood vessels, can migrate to distant organs. The content of CTCs in peripheral blood is extremely low, and thus detecting them requires a very sensitive methodology. SERS sensors for detecting CTCs consist of a plasmonic metal, specific DNA strands complementary to the mutated DNA from those cells, and sometimes attached RRM used to amplify the SERS signal. Zhang et al. (2019b) developed a nanosensor by assembling gold nanoparticles in triangular pyramidal DNA, which provides more hot spots and thereby enhances the SERS signal compared with standard nanoprobes. They were able to detect epithelial cell adhesion molecules, present on the CTC surface but absent from blood cells, at a concentration of 5 cells/ml. Moreover, it is possible to monitor several markers on CTCs simultaneously, which is much easier using SERS than, for example, fluorescence, where the multiplexing is limited by the number of channels in the microscope (maximum four). For example, anti-epithelial cell adhesion molecules, anti-CD44, anti-Keratin1820, and anti-insulin-like growth factor antigen have been detected on a MCF7 breast cancer CTCs without any enrichment, separation or other tedious and time-consuming procedures (Nima et al., 2014), showing the enormous potential of SERS, since as many as five different targets can be monitored without signal overlapping (Sánchez-Purrà et al., 2018).

#### Intracellular Environment

Apart from monitoring specific markers on the cellular membrane, SERS can be used to monitor the intracellular environment. It has been applied to measure, for example, the concentration of different ions (Mg^2+^ and Ca^2+^), pH, and redox potential. Usually, these values are measured indirectly by measuring the SERS spectra of specific reporter molecules, which change as a function of the concentration of the species of interest. For example, this approach was applied to monitor microRNA-21 and telomerase in cells (Liu et al., 2021). In this rather complicated procedure, the presence of telomerase weakens the signal from the RRM by triggering the dissociation of nanoprobes and releasing the reporter. In contrast, the target microRNA-21 can trigger the catalytic hairpin assembly amplification, leading to an enhancement of the signal of another reporter.

The SERS nanosensors can be successfully applied for measuring intracellular pH, even in selected organelles (lysosomes, nuclei, and mitochondria). Plasmonic metal nanoparticles labeled with pH-sensitive molecules and specific organelle-targeting peptides can be easily delivered into organelles, and the SERS mapping of the cell provides information about the pH in a specified location (Shen et al., 2018). These experiments are valuable, as there are no commercially available fluorescent indicators for measuring pH in mitochondria or the nucleus, and NMR-based or microelectrode-based measurements are still not routinely used because of their pitfalls (Jaworska et al., 2021). Moreover, in addition to providing detailed information about pH in specific cellular compartments, this procedure can be used for phototherapy. For example, gold nanorods labeled with a pH-sensitive dye (cysteine-hydroxyl merocyanine) were used to monitor pH in lysosomes. Their localization was confirmed by the fluorescence measured from the dye, and laser irradiation caused metal heating to 60°C, resulting in a significant decrease in solid tumors in living mice, on which the sensor was ultimately tested (Wen et al., 2021). Li et al. (2018) applied this procedure for human breast tumor specimens by staining 5-μm thin sections with a mixture of four antibody-labeled SERS probes, successfully targeting cerbB2, oestrogen, progesterone, and epidermal growth factor receptors expressed in the tissues. In a very impressive work by Wang et al. (2019), SERS was applied in order to measure $CO_{3}^{2-}$ and pH in living brains and neurons (Wang et al., 2019). Quartz tapers covered with gold and modified with 1-(4-aminophenyl)-2,2,2-trifluoroethanone (sensitive to $CO_{3}^{2-}$) and 4-mercaptobenzoic acid (sensitive to pH changes) were used as the SERS substrates. They were able to monitor changes in single neurons and in the cortex of a living mouse brain. This work provided a novel methodology for the development of biosensors for the simultaneous determination of multiple biological species in living systems and showed the potential of the SERS spectroscopy for challenging *in vivo* experiments.

#### Surface-Enhanced Raman Scattering Detection of Bacteria

Another interesting example of the potential of SERS is the detection of bacteria. Bi et al. (2020) developed a specific platform to detect *Escherichia coli* and test antibiotic susceptibility. With the use of rhodamine 6G-modified gold core–silver shell nanorods, they were able to detect bacteria at a level of 10^2^ CFU/ml to confirm their presence in spiked mouse blood and to discriminate antibiotic resistance within just 3.5 h. Apart from ultrasensitive detection of bacteria, in SERS-based approach, it is possible to go further and to photothermally kill bacteria (Lin et al., 2014). In their work, Lin et al. conjugated bacteria to the graphene oxide-modified tags, SERS detected both Gram-positive (*S. aureus*) and Gram-negative (*E. coli*) bacteria and, additionally, photothermally annihilated them, showing the potential of *in situ* monitoring of the photothermal antibacterial response using a SERS assay.

#### Surface-Enhanced Raman Scattering Combined With Other Methods

**Figure 2:**
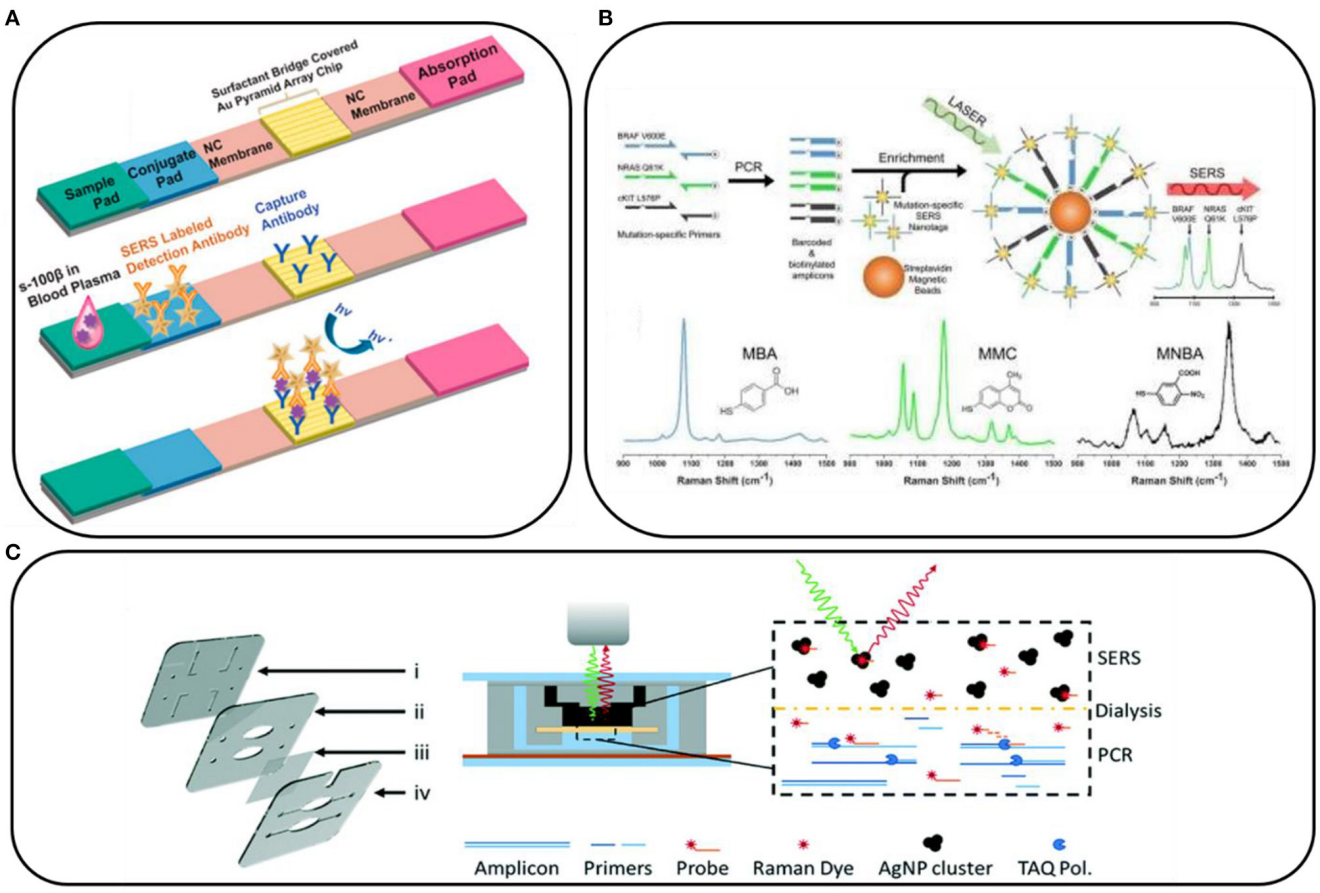
**(A)** Schematic illustration of a paper-based lateral flow strip. Reprinted with permission from Gao et al. (2021b). Copyright (2021) American Chemical Society; **(B)** Conceptual schematic of the multiplex PCR/surface-enhanced Raman spectroscopy (SERS) assay and SERS nanotags with the examples of Raman reporter molecules. Reprinted with permission from Wee et al. (2016). Copyright (2016) Ivyspring International Publisher; **(C)** Scheme of dialysis-driven PCR–SERS device. Reprinted with permission from Restaino and White (2018).

Interesting examples showing the potential of SERS in clinical applications include those in which SERS is combined with lateral flow assays. With this approach, the experimental procedure involves the preparation of strips, similar to pregnancy tests, which contain specific aptamers, antibodies, antigens, and SERS substrates that interact with the sample. Then, the appearance of the characteristic SERS signal confirms the presence of specific markers (Wang et al., 2017, 2018; Zhang et al., 2018; Gao et al., 2021a,b; Kim et al., 2021). Figure 2A shows an example of a paper-based lateral flow strip (PLFS) with an integrated plasma separation unit for the SERS detection of carcinoembryonic antigen (CEA) in 30 μl of whole blood, with a limit of detection of 1.0 ng/ml (Gao et al., 2021a). With this approach, many different compounds have been detected, for example, toxins, (prostate, kidney, and breast) cancer markers, HIV-1 DNA, bacterial pathogens, folic acid, α-fetoprotein, and others, as they have biological samples such as blood or blood plasma.

Also, the use of paper substrates significantly lowers the cost of the SERS substrates, especially when combined with microfluidics, where only a small volume of sample is needed. This approach was successfully applied by Torul et al. (2015) for sensing glucose in blood samples, which is one of the most intriguing topics emerging in the development of SERS. A combination of the described paper-based SERS-strips and low-cost portable Raman spectrometers could provide an opportunity for hospitals and diagnostic facilities to offer both a very low limit of detection and reasonable sample preparation and spectrometer costs.

The SERS spectroscopy is very often combined with PCR. PCR is used to amplify the target gene to a detectable level, and it is currently difficult to imagine detecting mutations in DNA and RNA without using it. For example, Lee et al. (2021a) combined PCR with SERS to prepare a paper-based sensor consisting of silver nanowires and Biotium's (San Francisco, USA) EvaGreen for the detection of bacterial DNA in the form of a point-of-care test. Also, DNA mutations in CTCs can be detected by the combination of these two techniques. For example, the tumor DNA is amplified by PCR and then tagged with mutation-specific SERS nanotags (Figure 2B). The mutation status is then evaluated using the Raman spectroscopy where unique spectral peaks indicate the presence of the mutation of interest (Wee et al., 2016). Scheme of dialysis-driven real-time PCR–SERS device used for the detection of methicillin-resistant *S. aureus* is shown in Figure 2C (Restaino and White, 2018).

## Conclusions and Future Perspectives

In this mini-review, we briefly outlined recent advances in the application of SERS for the detection and determination of certain biochemically (and especially medically) important species. We expect further intensive development of biochemical and chemical SERS sensors, especially DNA/RNA ones that can be used for the early identification of cancer-connected DNA mutations and various bacteria and viruses. Moreover, DNA sensors developed for medical applications may be further applied in other areas, for example, to verify the authenticity of some food products. While many working SERS biosensors have already been developed, many aspects still need to be significantly improved before commercialization can occur, for example, improving the reproducibility of SERS substrates. We hope that the examples presented in this mini-review will convince readers that the SERS spectroscopy is a very promising option for some of the routine analytical techniques used in medical, biochemical, and biological analyses.

## Author Contributions

AK and AS carried out literature searching and wrote the manuscript. All authors contributed to the manuscript and approved it for publication.

## Conflict of Interest

The authors declare that the research was conducted in the absence of any commercial or financial relationships that could be construed as a potential conflict of interest.
