# Supplementary material for: Applications of Surface-Enhanced Raman Scattering in Biochemical and Medical Analysis
Source: Front Chem. 2021 May 7;9:664134. doi: 10.3389/fchem.2021.664134 (PMC8138180; doi:10.3389/fchem.2021.664134)
Supplement: Supplementary file 1 [file Data_Sheet_1.PDF]

Applications of SERS in biochemical and medical analysis

Aleksandra Szaniawska[1*] and Andrzej Kudelski[1*]

[1]*Faculty of Chemistry, University of Warsaw, 1 Pasteur St., 02-093 Warsaw, Poland*

Surface enhanced Raman spectroscopy (SERS)

In 1970's it was observed that Raman signal generated by molecules adsorbed on some nanostructured materials is increased by many orders of magnitude (Jeanmaire and van Duyne, 1977; Albrecht and Creighton, 1977). This phenomenon was called surface-enhanced Raman scattering (SERS) and was explained as a result of the synergistic cooperation of two mechanisms: (i) based on excitation of the localized surface plasmons and (ii) based on the chemical interactions.

When nanostructures formed from materials with a negative real and a small positive imaginary dielectric constant at a given excitation frequency interact with an electromagnetic wave, the electric field of the excitation radiation induces collective oscillations of surface conduction electrons called surface plasmons (Aroca, 2006). Such oscillations can be viewed as the appearance of an oscillating electric dipole inducing an additional electric field in close proximity to the illuminated nanostructure. For a spherical metal nanoparticle the magnitude of the induced dipole (p) is proportional to:

$$p \sim \frac{\epsilon_M(\nu) - \epsilon_{out}(\nu)}{\epsilon_M(\nu) + 2\epsilon_{out}(\nu)}$$

where: ν is the frequency of the excitation radiation and $\epsilon_M(\nu)$ and $\epsilon_{out}(\nu)$ are the dielectric functions of the metal and the surrounding medium, respectively (Etchegoin and Ru, 2010; Aroca, 2006). For some combinations of the material and excitation radiation frequency used (for example, silver for any visible excitation radiation, or gold for the red excitation radiation), the value of this fraction is very large, which means that locally a strong electric field is induced. In the case of homogeneous plasmonic nanostructures, the strongest enhancement of the electromagnetic field occurs at the sharp apexes and edges; in a case of agglomerates or aggregates of plasmonic nanostructures very large field enhancement is observed in the slits between nanograins - such places are called “hot spots” (Hao and Schatz, 2004). In SERS spectroscopy the increase in the efficiency of the Raman signal generated is roughly proportional to the fourth power of the field enhancement (Aroca, 2006; Kudelski, 2009), and because of this fourth power dependence, very large SERS enhancement factors can be achieved, making SERS spectroscopy one of the most sensitive analytical tools.

The chemical mechanism of SERS involves the hybridization of orbitals of the adsorbed molecules with the orbitals of metal, which facilitates resonance Raman scattering. Since in the

electrochemical systems the energy of the electronic levels in metal can be changed by the variation of the potential drop across the interface, one can tune the system into or out of the resonance conditions not only by changing the wavenumber of the excitation radiation but also by changing the applied potential. The chemical mechanism is only important for molecules interacting directly with the metal surface, and therefore, is not operating in many SERS sensors.

References

Albrecht, M. G., and Creighton, J. A. (1977). Anomalously intense Raman spectra of pyridine at a silver electrode. *J. Am. Chem. Soc.* 99, 5215–5217. doi: 10.1021/ja004 57a071.

Aroca, R. (2006). *Surface-Enhanced Vibrational Spectroscopy*. John Wiley & Sons.

Etchegoin, P. G., and Ru, E. C. L. (2010). "Basic Electromagnetic Theory of SERS," in *Surface Enhanced Raman Spectroscopy* (John Wiley & Sons, Ltd), 1–37. doi:10.1002/9783527632756.ch1.

Hao, E., and Schatz, G .C. (2004). Electromagnetic fields around silver nanoparticles and dimers. *J. Chem. Phys.* 120, 357–366. doi: 10.1063/1.16292 80

Jeanmaire, D. L., and Van Duyne, R. P. (1977). Surface Raman spectroelectrochemistry part I. Heterocyclic, aromatic, and aliphatic amines adsorbed on the anodized silver electrode. *J. Electroanal. Chem.* 84, 1–20. doi:10.1016/S0022-0728(77)80224-6

Kudelski, A. (2009). Raman spectroscopy of surfaces. *Surf. Sci.* 603, 1328–1334. doi: 10.1016/j.susc.2008.11.039.
